# Laparoscopic and Endoscopic Cooperative Surgery for Gastric Submucosal Tumor Near Esophagogastric Junction With Sliding Hiatal Hernia

**DOI:** 10.7759/cureus.37902

**Published:** 2023-04-20

**Authors:** Hajime Kashima, Satoru Kikuchi, Shinji Kuroda, Toshiyoshi Fujiwara

**Affiliations:** 1 Department of Gastroenterological Surgery, Okayama University Graduate School of Medicine, Dentistry and Pharmaceutical Sciences, Okayama, JPN

**Keywords:** lecs, local resection, esophagogastric junction, hiatal hernia, laparoscopic surgery, leiomyoma, gastric submucosal tumor

## Abstract

The usefulness of laparoscopic and endoscopic cooperative surgery (LECS) for gastric submucosal tumors in the cardiac region has been reported in recent years. However, LECS for submucosal tumors at the esophagogastric junction with hiatal sliding esophageal hernia has not been reported, and its validity as a treatment method is unknown. The patient was a 51-year-old man with a growing submucosal tumor in the cardiac region. Surgical resection was indicated because a definitive diagnosis of the tumor was not determined. The lesion was a luminal protrusion tumor, located on the posterior wall of the stomach 20 mm from the esophagogastric junction, and had a maximum diameter of 16.3 mm on endoscopic ultrasound examination. Because of the hiatal hernia, the lesion could not be detected from the gastric side by endoscopy. Local resection was considered to be feasible because the resection line did not extend into the esophageal mucosa and the resection site could be less than half the circumference of the lumen. The submucosal tumor was resected completely and safely by LECS. The tumor was diagnosed as a gastric smooth muscle tumor finally. Nine months after surgery, a follow-up endoscopy showed reflux esophagitis. LECS was a useful technique for submucosal tumors of the cardiac region with hiatal hernia, but fundoplication might be considered for preventing backflow of gastric acid.

## Introduction

Recently, the usefulness of local excision by laparoscopic and endoscopic cooperative surgery (LECS) for submucosal tumors in the region of gastric cardia has been reported, and it has become one of the treatment options [1]. However, the validity of LECS for patients with sliding hiatal hernia in which the tumor site is elevated above the hiatus as a treatment method is still unclear, as there are few reports so far. We have recently experienced a case of gastric leiomyoma in the region of the fundus with a hiatal hernia and the patient was indicated for surgical resection. In this case, we demonstrate the technique of LECS for gastric submucosal tumors located near the esophagogastric junction with sliding hiatal hernia and assessed the feasibility of treatment.

## Case presentation

The patient is a 51-year-old male, height 159.9 cm, and weight 67.8 kg. He had a gastric submucosal mass with a diameter of 10 mm at a medical checkup seven years earlier. The mass was followed up by ultrasound endoscopy every year and did not change in size, but this time, it was 16.3 x 14.7 mm in diameter and showed a tendency to increase. Thus, the patient underwent a thorough examination including fine needle aspiration under endoscopic ultrasonography.

**Figure 1:**
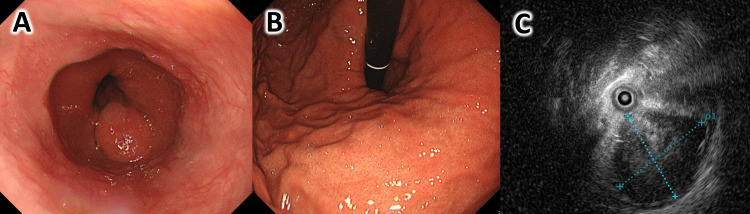
Preoperative upper gastrointestinal endoscopic and ultrasound image (A) View from the esophageal side. The esophagogastric junction is elevated toward the thoracic cavity due to a hiatal hernia, and the lesion is located on the posterior wall of the esophagogastric junction region. (B) View from the gastric side. The lesion located above the diaphragmatic hiatus cannot be seen from the gastric lumen by the endoscope. (C) Ultrasound image. The lesion is 16.3 x 14.7 mm in diameter, with a hypoechoic area near the margins and mosaic-like echo intensity in the center.

Upper gastrointestinal endoscopy revealed a hiatal hernia with a sliding esophageal hiatal hernia. The mass was located on the posterior wall just below the esophagogastric junction and was a lumen protruding lesion. It could be localized from the esophageal side but was not visible from the gastric lumen due to a hiatal hernia (Figure 1A, 1B). Ultrasonography showed mostly hypoechoic areas but some mosaic-like echo intensity near the center (Figure 1C). The distance from the esophagogastric junction was 20 mm, and the elevated area was less than half the circumference of the esophagogastric junction.

**Figure 2:**
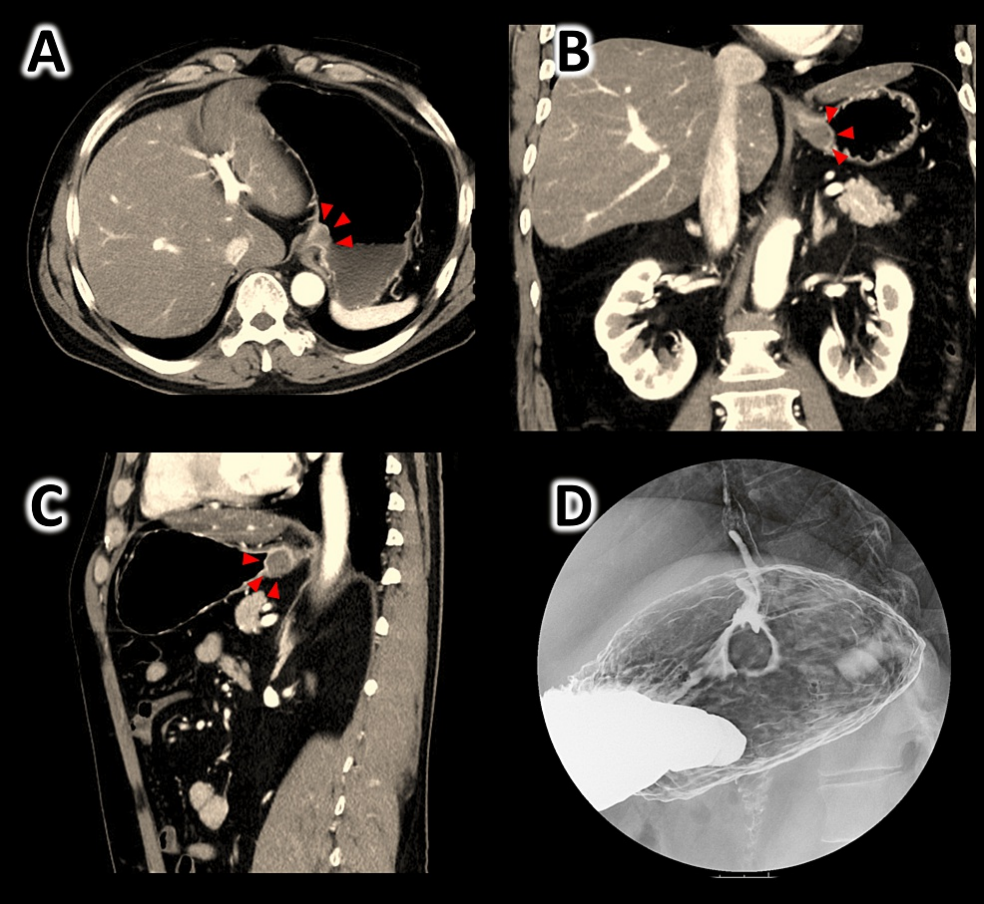
Preoperative thoracoabdominal contrast-enhanced CT image and X-ray image Red arrowheads show the lesion. (A) CT image, horizontal section. (B) CT image, coronal section. (C) CT image, sagittal section. (D) Contrast-enhanced X-ray image shows a 2-cm mass lesion just below the esophagogastric junction.

CT findings: A 17-mm large mass with well-defined margins was observed in the gastroesophageal junction (Figure 2A-2C). Contrast-enhanced X-ray image of the upper gastrointestinal tract: The lesion was depicted as a marginal well-defined shadow defect of approximately 20 mm in size just near the esophagogastric junction (Figure 2D).

**Figure 3:**
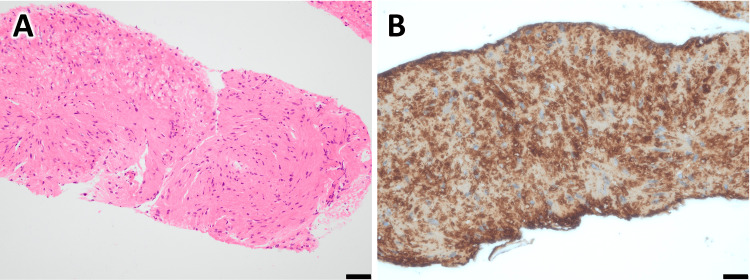
Histology from fine needle aspiration sample (A) HE staining shows spindle-shaped cells with deposition of acidophilic material. (B) Immunohistochemistry shows α-SMA-positive cells. Scale bar: 50μm.

Histopathology: Hematoxylin and eosin (HE) staining showed that the majority of the lesion consisted of spindle-shaped cells with deposition of acidophilic material (Figure 3A). Immunohistochemical staining was weakly positive for c-kit, but alpha-smooth muscle actin (α-SMA) was positive, and leiomyoma was the first differential diagnosis (Figure 3B). However, additional DOG-1 staining was also partially positive, and gastrointestinal stromal tumor (GIST) could not be ruled out. Based on the above, we decided to perform a surgical resection and chose a partial gastrectomy with LECS.

**Figure 4:**
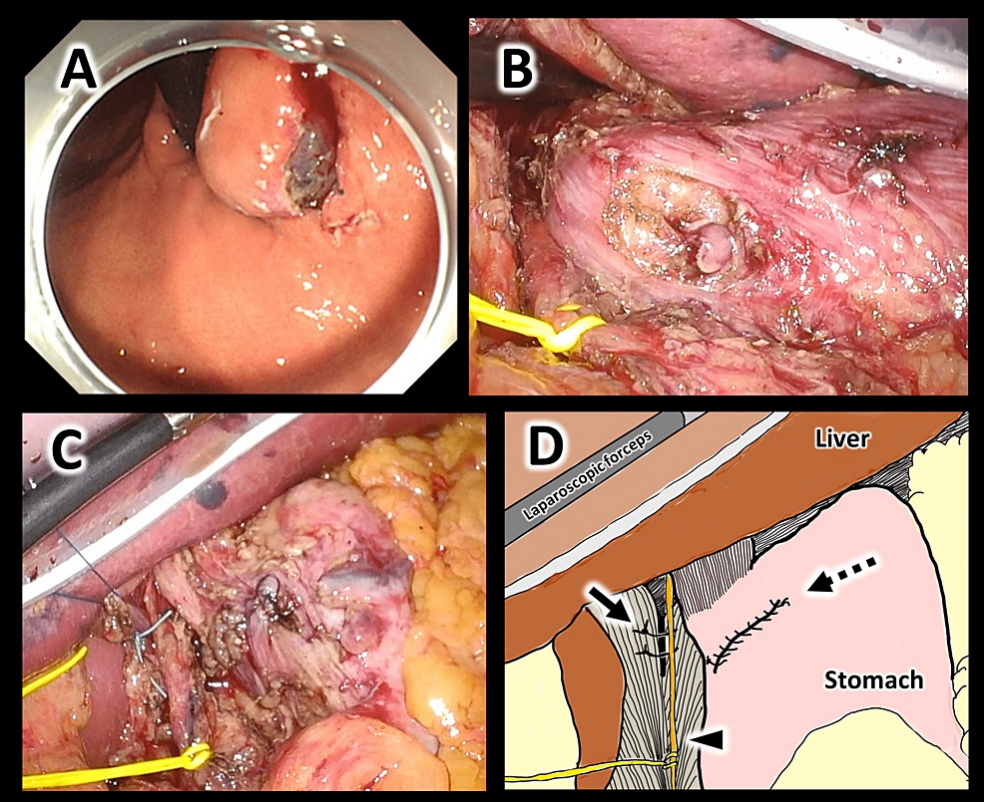
Intraoperative findings (A) The lesion can be observed from the gastric side after dissection of the gastric mesentery and traction of the stomach. (B) The gastric wall was perforated from the endoscope side on the caudal side of the lesion. The vagus nerve was separated with yellow tape. (C) Intraoperative laparoscopic view after suturing the serosal muscle layer and the diaphragmatic crus. (D) Schematic illustration after resection and suturing. The solid arrow shows sutured diaphragm crus. The dashed arrow shows the sown line of the gastric wall. The arrowhead shows the vagus nerve retracted by yellow tape.

Surgical findings: Under general anesthesia, the patient was placed in the open leg position. Five ports were placed at the umbilicus, right hypochondrium, right side of the abdomen, left hypochondrium, and left side of the abdomen. The extrahepatic area was elevated with a silicone disk to secure the field of view. First, the periampullary mesentery was treated, and the esophagogastric junction area was dissected from the diaphragmatic leg in preparation for resection of the lesion. The posterior trunk of the vagus nerve was taped and preserved. After dissection of the mesentery around the esophagogastric junction, the lesion could be easily identified by endoscopic inversion from the gastric side. An endoscopic submucosal incision around the mass was made (Figure 4A). The gastric wall was then endoscopically perforated through the incision site on the distal side of the tumor while observing the gastric wall through the laparoscope. A full-layer incision was made along the line of the submucosal incision from the abdominal side using a laparoscopic coagulation system (Figure 4B). After resection, the site of the gastric wall defect was closed by continuous suture of the submucosal layer with 4-0 PDS and continuous suture of the serosal muscular layer with 3-0 V-Loc. The open diaphragmatic crus after dissection was sutured with two stitches with 2-0 Ethibond (Figure 4C, 4D). An endoscope was inserted to confirm that there was no obstruction or bleeding. The operation time was four hours and 43 minutes with 100 mL blood loss.

**Figure 5:**
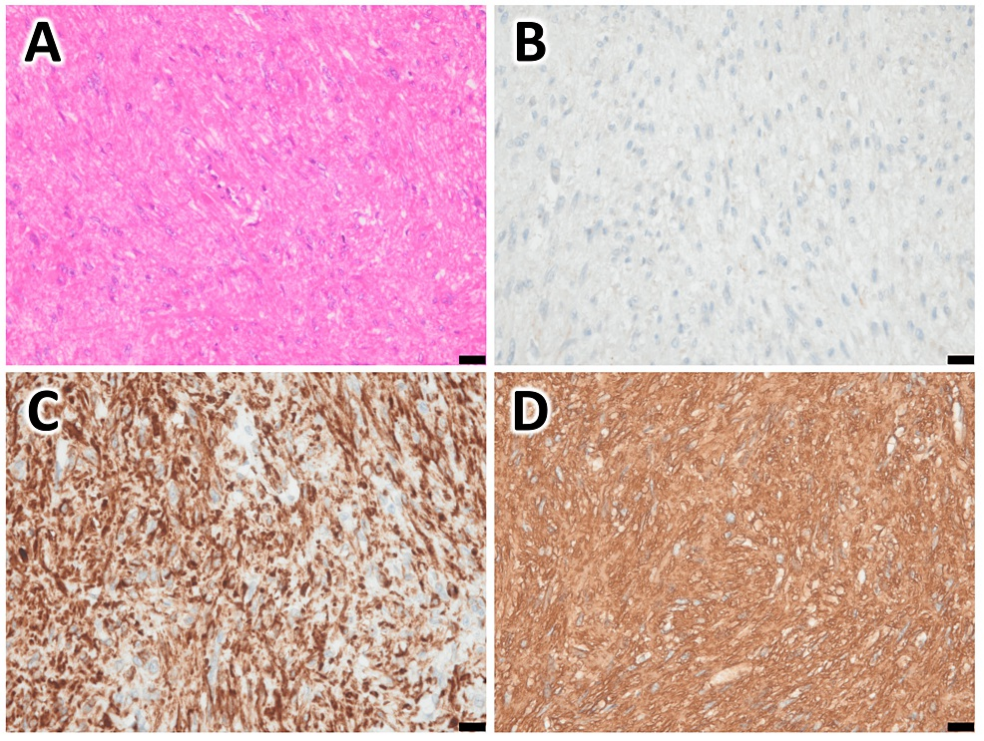
Histology from a surgical specimen (A) HE staining. (B) Immunohistochemistry for c-kit shows negative. (C) IHC for desmin shows positive. (D) IHC for α-SMA shows positive. Scale bar: 20μm.

Postoperative pathology: HE staining showed spindle-shaped cells like a preoperative aspiration sample (Figure 5A). Immunohistochemical staining was negative for c-kit and DOG-1 and positive for α-SMA and desmin, and the lesion was finally diagnosed as gastric leiomyoma (Figure 5B-5D).

**Figure 6:**
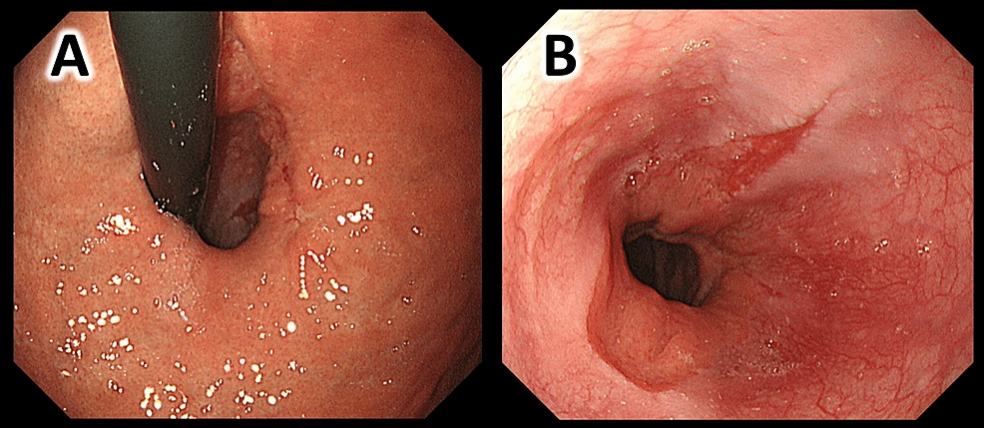
Endoscopic image nine months after surgery (A) View from the gastric side. (B) View from the esophageal side.

Postoperative course: Fluid intake was started the day after surgery, a liquid diet was started on the third day, and the diet had been transitioned to ordinary food day by day. The patient started to eat a regular diet on day seven. He was discharged on the ninth postoperative day. An endoscopic survey was performed nine months after the surgery and showed hiatal hernia and reflux esophagitis graded Los Angeles B (Figure 6A, 6B). The patient had no symptoms associated with the upper gastrointestinal tract.

## Discussion

In this case, LECS was useful for a gastric submucosal tumor in the esophagogastric junction region associated with a sliding hiatal hernia technically. However, adding fundoplication after local excision might be considered to prevent the recurrence of hiatal hernia and reflux esophagitis.

According to the diagnostic algorithm of GIST practice guidelines, patients with resectable localized gastrointestinal submucosal tumors that are asymptomatic, biopsy-undiagnosed, and less than 2 cm in size are basically followed up [2]. A tumor that had remained unchanged at 10 mm in size for seven years was found to have increased to 16.3 mm in length at the present examination. We determined that this was a case with a relative indication for surgery and performed a fine needle aspiration under endoscopic ultrasonography (EUS-FNA) to first make a histological diagnosis.

EUS-FNA is often sufficient to obtain a specimen for a gastric submucosal mass lesion, and a diagnosis can be made by combining HE staining and immunohistochemical staining [3]. In this case, HE staining showed bundles of tumor cells with acidophilic deposits, which suggested a spindle cell type of GIST [4]. However, additional immunostaining for c-kit was partially positive and difficult to determine. Thus, α-SMA staining was added to confirm its positivity. In addition, GIST is the most common submucosal tumor in the stomach as a whole, at about 70%, but when limited to the esophagogastric junction region, smooth muscle tumors are reported to be more frequent than GIST, at 86% [5,6]. Based on these data, leiomyoma was also mentioned as the first differential. The data also indicated that leiomyoma was the first differential diagnosis. However, we could not rule out GIST because α-SMA-positive GISTs also occur in about 30-40% of cases [7]. Furthermore, DOG-1 staining is recommended in cases where the diagnosis is not clear, and we performed DOG-1 staining as an additional test, but like c-kit, it was difficult to determine, and the possibility of GIST could not be ruled out [8]. Therefore, the decision was made to perform surgical resection after consultation with the patient because, as mentioned above, a lesion showing an increasing trend toward growth is a relative indication for surgery according to the guidelines. Besides, the reason for the decision to operate at this stage was the possibility that a growing submucosal tumor, even a benign tumor such as leiomyoma, might not be resected locally and would require a more invasive operation if operated on after the tumor had grown and symptoms such as impaired transit and the bleeding had appeared.

For gastrointestinal submucosal tumors for which a preoperative diagnosis cannot be made, a partial resection with a reserved resection margin is performed based on the GIST treatment plan. In the past, gastric submucosal tumors were often treated by laparoscopic wedge resection using an automated suture device [9,10]. However, since the LECS was reported in 2008, its usefulness has been widely recognized [11]. Since it is impossible to confirm the location of the tumor from the laparoscopic side in a conventional local resection, this method, which penetrates the stomach wall while confirming the location of the tumor from the lumen, can ensure resection of the lesion. Simply resecting from the abdominal cavity while confirming the location of the tumor may result in excess resection of the gastric wall or in an incision line that is too close to the esophagogastric junction. To prevent this, endoscopic tattooing from the gastric wall is very useful. This allows for local resection in cases that would have previously been treated with proximal gastrectomy. Multicenter data from Japan also report that LECS is a safe and feasible procedure for gastric submucosal tumors [1]. However, caution should be exercised when the lesion is nearly located in the esophagogastric junction region. If the resection line is close to the esophagogastric junction, it may be difficult to preserve the function of the fundus and there is a risk of postoperative stenosis. Therefore, the surgical decision should be made carefully. Some reports suggest that LECS for submucosal tumor lesions in the esophagogastric junction region is a risk factor for open conversion and that preoperative preparation should be made to safely convert the lesion to a proximal gastrectomy [12]. Especially in cases where the lesion extends into the esophagus, open conversion or laparoscopic proximal gastrectomy was performed in a high percentage of patients. The number of patients who underwent laparotomy or laparoscopic gastrectomy was high. In this case, the endoscopic findings indicated that the resection area was less than half the circumference of the esophagus and that suture closure could be achieved safely and accurately laparoscopically. Therefore, we decided to perform a local resection. Since the distance from the esophagogastric junction to the rise of the oral ridge was approximately 2 cm, the resection area was not considered to extend into the esophagus, but we were prepared for a change in the surgical technique before proceeding to surgery.

**Figure 7:**
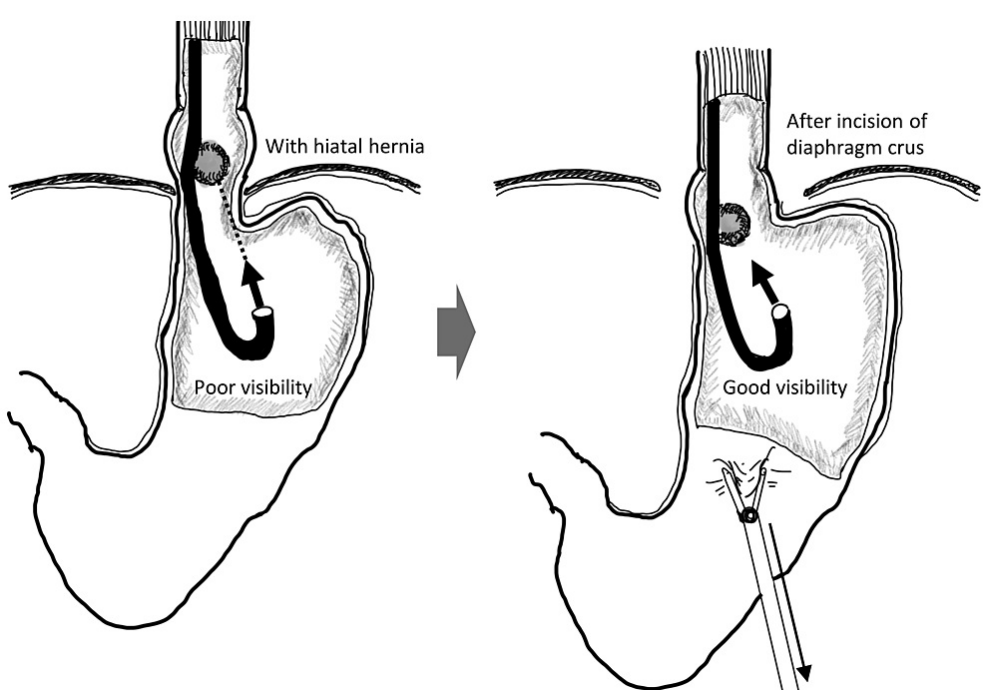
Location of the diaphragmatic leg and esophagogastric junction before and after peridiaphragmatic dissection

Furthermore, this case was accompanied by a slipped hiatal hernia of the esophagus. Thus, more ingenuity was required to ensure safe and reliable resection of the lesion. Although various techniques were used in the previous gastric submucosal tumor cases, there were no reports of cases with esophageal hiatal hernias. In LECS, it is of course important to have a good view laparoscopically, but it is also important to have a good view endoscopically as well. The lesion was difficult to see through the stomach preoperatively due to a hiatal hernia, but a good endoscopic view could be obtained by laparoscopic dissection of the esophagogastric junction (Figure 7).

Asymptomatic hiatal hernia without gastroesophageal reflux disease (GERD) is not usually treated surgically. According to the guidelines for the treatment of GERD, medication is the first step even in the presence of symptoms [13]. In the present case, the patient was asymptomatic despite the presence of a preoperative hiatal hernia with a slipped esophageal hiatus.

However, we had to carefully consider the procedures for treatment. Because even if the patient had no symptoms before surgery, the diaphragm crus were dissected and separated from the gastrointestinal tract. As a result, it made gastric cardia looser. We decided to suture the diaphragm crus with a non-absorbable thread in order to reduce the risk of clinical manifestations associated with dissection around the diaphragmatic crus. Due to the lack of a related report, we were not sure whether to add fundoplication or not. However, because the original hernia was minor, we have not performed a fundoplication in this case. The results suggest that it may have been better to add fundoplication, but since this is a single case study, further case series are warranted to determine whether fundoplication is necessary after local resection of submucosal tumors in the esophagogastric junction region with esophageal hiatus herniation.

## Conclusions

We report a LECS technique for patients with sliding hiatal hernia in which the tumor site is located near the esophagogastric junction.

LECS was technically useful as a treatment approach for a gastroesophageal submucosal tumor associated with a sliding hiatal hernia. However, we might consider adding fundoplication for preventing the recurrence of hiatal hernia and reflux esophagitis.
